# The ED_50_ and ED_95_ of remimazolam tosilate to inhibit the gastroscopy reaction in elderly patients of varying ages: an up-and-down sequential allocation trial

**DOI:** 10.3389/fmed.2025.1489771

**Published:** 2025-05-15

**Authors:** Yang Yang, Hao-Jing Xiong, He-Chen Xu, Hong-Qi Wu, Mi Chen, Ke Jiang, Xiao-Hua Zou

**Affiliations:** ^1^Department of Anesthesiology, the Affiliated Hospital of Guizhou Medical University, Guiyang, China; ^2^College of Anesthesiology, Guizhou Medical University, Guiyang, China

**Keywords:** remimazolam, elderly, gastroscopy, geriatric, effective dose, up-and-down method

## Abstract

**Objective:**

Although remimazolam tosilate (RT) has demonstrated efficacy in elderly gastroscopy, its optimum dose for gastroscopy in elderly patients of varying ages is still unclear. The study aimed to determine the median effective dose (ED_50_) and 95% effective dose (ED_95_) of RT to inhibit the gastroscopy reaction in patients aged 65–74 years and 75–89 years.

**Methods:**

Sixty-one elderly patients were randomized into two groups: Group A (65–74 years, *n* = 31) and Group B (75–89 years, *n* = 30). Three minutes after the recruited subjects received 0.1 μg/kg of sufentanil, RT was administered intravenously at an initial dose of 0.15 mg/kg with 0.01 mg/kg step size according to the modified Dixon’s up-and-down method (UDM). This continued until nine crossover midpoints “positive–negative” were observed.

**Results:**

The ED_50_ of RT calculated by the modified UDM for inhibiting the gastroscopy reaction in Group A (0.175 (95% confidence interval, CI, 0.166–0.183) mg/kg) was higher than that in Group B (0.163 (95% CI, 0.156–0.169) mg/kg) (*p* = 0.03). The ED_95_ estimated by probit regression analysis was 0.199 (95% CI, 0.186–0.244) mg/kg in Group A and 0.188 (95% CI, 0.175–0.232) mg/kg in Group B.

**Conclusion:**

RT is a relatively safe sedative hypnotic that can provide a suitable sedative effect for elderly patients undergoing a gastroscopy.

**Clinical trial registration:**

http://www.chictr.org.cn, identifier ChiCTR2200062842.

## 1 Introduction

With the aging of the population and the popularization of comfortable medical treatment, the proportion of elderly patients undergoing gastroscopic sedation is increasing. However, the elderly patients are more sensitive to anesthetics due to reduced metabolic capacity and increased central nervous system responsiveness, weak tolerance of anesthesia, and the risk of anesthesia also increases (1–3). Thus, a moderate to deep degree of sedation is often required for gastroscopy, adverse events such as hypotension, respiratory depression, bradycardia, and intravenous pain are often caused by the commonly used anesthetics in current clinical practice, and even cardiopulmonary failure and emergency tracheal intubation may be induced. Moreover, the incidence of adverse events in elderly patients may be higher with increased age (4, 5). Remimazolam is a novel hydrosoluble, short-acting, intravenous benzodiazepine that causes inhibition of neuronal activity through the *γ*-aminobutyric acid subtype A (GABAA) receptor pathway (6, 7). The pharmacokinetic profile of remimazolam is characterized by a rapid onset of 1–3 min, a short terminal half-life of 10–20 min, and context-sensitive decrement times comparable to those of other short-acting hypnotic agents (7). With its properties such as non-irritating nature, rapid onset, short half-life, predictable sedation levels, swift emergence from anesthesia, kidney-and liver-independent metabolism, minimal residual sedation, negligible side effects at typical dosages (e.g., cardiovascular/respiratory depression), absence of adrenal cortex inhibition, myoclonic movements, or injection site pain, and preservation of mitochondrial activity and fatty acid oxidation, remimazolam emerges as a promising option for outpatient sedation (6–9). Studies have shown that RT has been effectively and safely used for gastroscopic sedation in young and middle-aged patients (10). Another study suggested that the effect of anesthetics might vary in different age groups by reason of the physiological degeneration in elderly patients (5). However, a more rigorous grading of evidence is essential to ensure methodological transparency and strengthen the validity of conclusions in future studies. The dose-effect relationship of RT during gastroscopic sedation in elderly patients of varying ages is not completely understood. To provide a reference for optimizing gastroscopic sedation in elderly patients of varying ages, an up-and-down sequential allocation trial was used to estimate the optimum doses and safety of RT.

## 2 Materials and methods

This prospective up-and-down sequential allocation trial was conducted in accordance with the Declaration of Helsinki, after Ethics Committee approval. Written informed consent was obtained prior to patient enrolment. The dose distribution was determined according to the modified Dixon’s up-and-down method (UDM) (11–13). The elderly patients, aged 65–89 years, who underwent gastroscopic sedation in the Affiliated Hospital of Guizhou Medical University from August 25 to October 31, 2022, were enrolled in this trial. Participants were randomized into two groups: Group A (aged 65–74 years) and Group B (aged 75–89 years). The inclusion criteria were as follows: (a) scheduled for gastroscopy; (b) aged 65–89 years, male or female; (c) with ASA physical status I-II; (d) BMI of 18.0–29.9 kg/m^2^; (e) patients clearly understand and voluntarily participate in the study, and sign the informed consent form themselves. The exclusion criteria were as follows: (a) undergo tracheal intubation or laryngeal mask; (b) with acute heart failure, unstable angina, myocardial infarction occurred within 6 months, resting ECG heart rate<50 beats/min (B/M), third-degree atrioventricular block, severe arrhythmia, moderate to severe heart valve disease; (c) suffering from severe respiratory or mental system diseases and long-term use of psychiatric drugs and cognitive dysfunction; (d) with expected difficult airway; (e) hemoglobin<9 g/dL, platelet<80 × 10^9^/L, systolic blood pressure (SBP) ≥ 160 mmHg or≤90 mmHg, and/or diastolic blood pressure (DBP) ≥ 100 mmHg before gastroscopy; (f) with a history of drug abuse or alcohol abuse within 2 years; (g) allergic or contraindicated to benzodiazepines, or opioids; (h) participated in other clinical trials within 3 months; (i) considered unsuitable for this trial by the researcher.

All patients did not receive any premedication and were required to routinely fast for no less than 8 h before the gastroscopy. Upon entering the operating room, the Lactate ringer’s solution was intravenously infused at 5 mL/kg/h with a 22G venous cannula placed, and the routine vital signs monitoring were performed and recorded, including the electrocardiogram (ECG), heart rate (HR), noninvasive blood pressure (NBP), pulse oximetry (SpO_2_) and respiratory rate (RR). Patients were preoxygenated with 100% oxygen at 4 L/min with a nasal mask. The levels of sedation and anesthesia were determined using the Modified Observer’s Assessment of Alertness/Sedation scale (MOAA/S), which was evaluated by an anesthesiologist who blinded to the study design.

According to the results of our previous preliminary work and the principle of modified UDM, 3 min after sufentanil citrate (0.1 μg/kg, Yichang, China) was administered intravenously (within 60 s), an initial dose of 0.15 mg/kg of RT was administered intravenously in both groups, and the evaluation of the MOAA/S score commenced immediately upon the initiation of the intravenous administration of RT, with consistent scoring until the MOAA/S score≤2 (responds only after mild prodding or shaking) (14) and endoscope was then immediately attempted to be inserted. Each patient’s response determined the dose of RT for the next patient as described by Dixon (11), and the step size of the dose of RT was set as 0.01 mg/kg. The gastroscopy reactions were defined as bucking, body movement, nausea and vomiting. The response of patients to the gastroscopy reactions was determined by another anesthesiologist blinded to the dose of RT as either positive or negative. Successful sedation (negative response) was defined as MOAA/S score≤2 without gastroscopy reaction within 5 min from endoscope insertion, resulting in a 0.01 mg/kg decrease for the next patient. In contrast, failed sedation (positive response) was defined as MOAA/S score>2 with gastroscopy reaction within 5 min from endoscope insertion, an additional 1/3 of the initial dose of RT would be given at least 1 min apart as a rescue sedation once the gastroscopy reaction obviously interfered with the endoscopist’s procedure, and followed by a 0.01 mg/kg increase for the next patient. If the anesthesiologist determined that RT failed to achieve adequate sedation and propofol was substituted as alternative treatment, the patient would be excluded from the study. The first case enrolled in this study was counted from the previous patient at the first crossover midpoint, and the crossover midpoint was defined as the case that changed from positive to negative response. The trial was stopped after at least nine crossover midpoints occurred, and at least 20 patients were enrolled in each group.

Hypotension was defined as SBP ≤ 80mmHg and/or decreased≥20% of the baseline, and was treated with intravenous administration of ephedrine 3–9 mg. Respiratory depression was defined as SpO_2_<90% and/or RR<8 times/min, and was treated with 100% oxygen assisted ventilation. HR<50 B/M was intravenously treated with atropine 0.2–0.5 mg.

**Figure 1:**
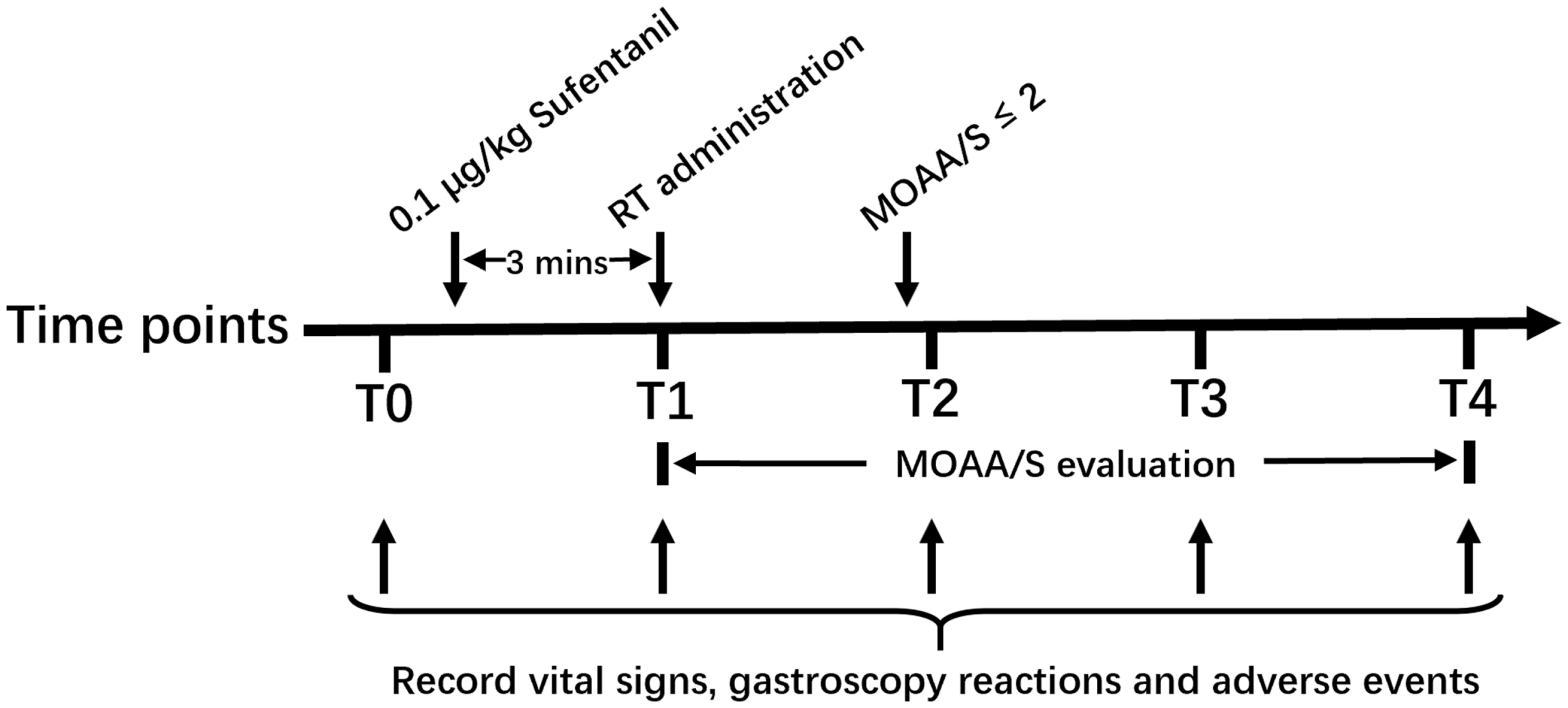
Graphical representation of protocol. T0, on arrival to the endoscopy room; T1, after RT administration; T2, after endoscope insertion; T3, 5 min after start of gastroscopy; T4, end of gastroscopy. RT, remimazolam tosilate; MOAA/S, the Modified Observer’s Assessment of Alertness/Sedation.

The vital signs (including NBP, HR, SpO_2_ and RR), gastroscopy reactions (including bucking, body movement, nausea and vomiting), and adverse events (including hypotension, respiratory depression, injection pain, arrhythmias, and muscle rigidity) were recorded at the following time points: on arrival to the endoscopy room (T0), after RT administration (T1), after endoscope insertion (T2), 5 min after the start of gastroscopy (T3), and the end of gastroscopy (T4). The graphical representation of protocol was shown in Figure 1. The induction time, sedation time of initial dose and gastroscopy time were recorded. The induction time was defined as duration from initial dose of RT administration to MOAA/s score≤2, and sedation time of initial dose was defined as duration from MOAA/s score≤2 induced by initial dose of RT to MOAA/S score>2, and the gastroscopy time was defined as duration from the gastroscope insertion to exit.

SPSS 18.0.0 software (IBM SPSS Statistics Inc., New York, USA) was used for statistical analysis, and GraphPad Prism 9.3.1 (GraphPad Software Inc., San Diego, CA, USA) was performed for drawing figures. In the case of normal data distribution, mean (standard deviation, SD) was used, and the comparison between the two groups was conducted using the independent sample *t*-test. In the case of non-normal data distribution, median [interquartile range, IQR] was used, and the comparison between the two groups was performed using the Mann–Whitney *U* test. Categorical data were expressed as numbers and proportions (%), and the comparisons between groups were analyzed by the *χ*2 test or Fisher’s exact probability test. The data of median effective dose (ED_50_) and 95% effective dose (ED_95_) were expressed as the mean (95% CI). The ED_50_ values were determined using both the modified UDM and probit regression analysis. The ED_95_ values were estimated by the probit regression analysis. Since the ED_50_ values of the two groups did not all follow a normal distribution, the comparison of ED_50_ values between the two groups was conducted using the Mann–Whitney *U* test. *p*<0.05 was considered statistically significant.

## 3 Results

**Figure 2:**
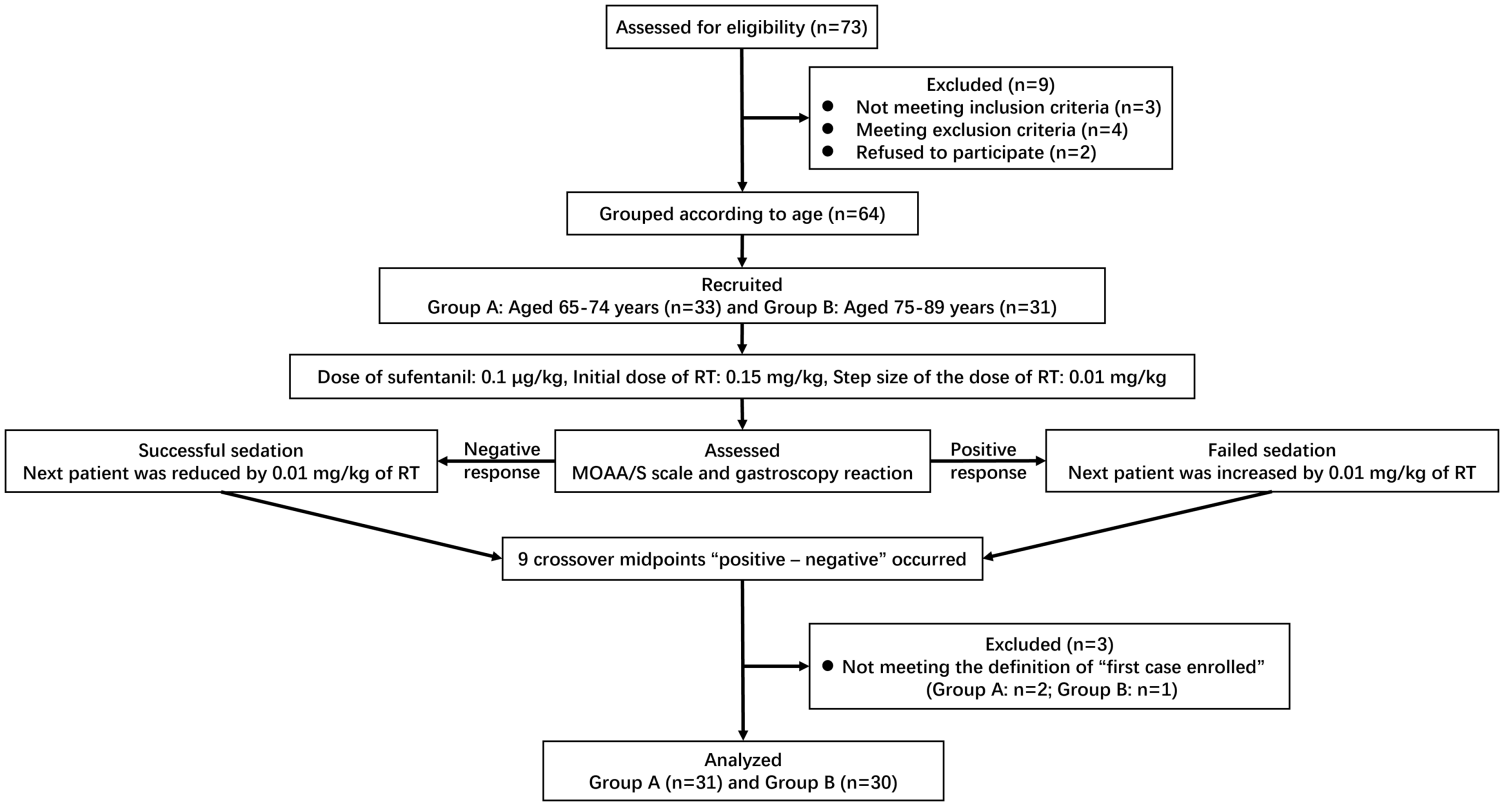
Flow diagram for the Dixon’s up-and-down method of included participants. RT, remimazolam tosilate; MOAA/S, the Modified Observer’s Assessment of Alertness/Sedation.

A total of 73 elderly patients were screened during the study. Seven patients did not meet the inclusion criteria, or met the exclusion criteria. Two patients refused to participate and withdrew their informed consent. None of the patients received propofol as an alternative treatment. All 33 and 31 patients completed the trial in Group A and Group B, respectively. Finally, 31 patients in Group A and 30 in Group B were enrolled in this study according to the above definition of the “first case.” The flow diagram of this trial was shown in Figure 2. Patient baseline demographics and clinical characteristics were listed in Table 1. There was no significant difference in baseline demographics between the two groups except for age (*p*<0.001). Most of the 61 patients enrolled had medical comorbidities, but there was no difference with respect to hypertension, diabetes, ischemic heart disease and sinus bradycardia between the two groups (*p*>0.05). No significant difference was observed in the induction time, sedation time of initial dose and gastroscopy time between the two groups (*p*>0.05).

**Table 1:** Baseline demographics and clinical characteristics of patients included.

Characteristic	Group A (*n* = 31)	Group B (*n* = 30)	*p*
Age (yr), mean (SD)	69.4 (3.1)	78.8 (3.4)	<0.001*
Male sex, *n*/total (%)	15/31 (48.4%)	17/30 (56.7%)	0.517
ASA physical status, *n*/total (%)			
I	13/31 (41.9%)	14/30 (46.7%)	0.710
II	18/31 (58.1%)	16/30 (53.3%)	
Weight (kg), mean (SD)	57.2 (9.8)	54.6 (9)	0.280
Height (cm), mean (SD)	158.3 (8.7)	157.6 (11.1)	0.797
BMI (kg/m^2^), mean (SD)	22.8 (3)	22.0 (2.6)	0.292
Medical comorbidities, *n*/total (%)			
Hypertension	20/31 (64.5%)	14/30 (46.7%)	0.161
Diabetes	2/31 (6.5%)	4/30 (13.3%)	0.425
Ischemic heart disease	1/31 (3.2%)	3/30 (10%)	0.354
Sinus bradycardia	5/31 (16.1%)	5/30 (16.7%)	1.000
Induction time (s), median [IQR]	58 [50–76]	53 [46.8–62]	0.121
Sedation time of initial dose (min), median [IQR]	3.3 [1.2–6.6]	4.8 [1.4–9.9]	0.316
Gastroscopy time (min), median [IQR]	6.8 [4–13.3]	7.9 [5.5–10.8]	0.502

Group A, patients aged 65–74 years; Group B, patients aged 75–89 years. SD, standard deviation; ASA, American Society of Anesthesiologists; BMI, body mass index; IQR, interquartile range. *p < 0.05 was considered to indicate statistical significance.

**Figure 3:**
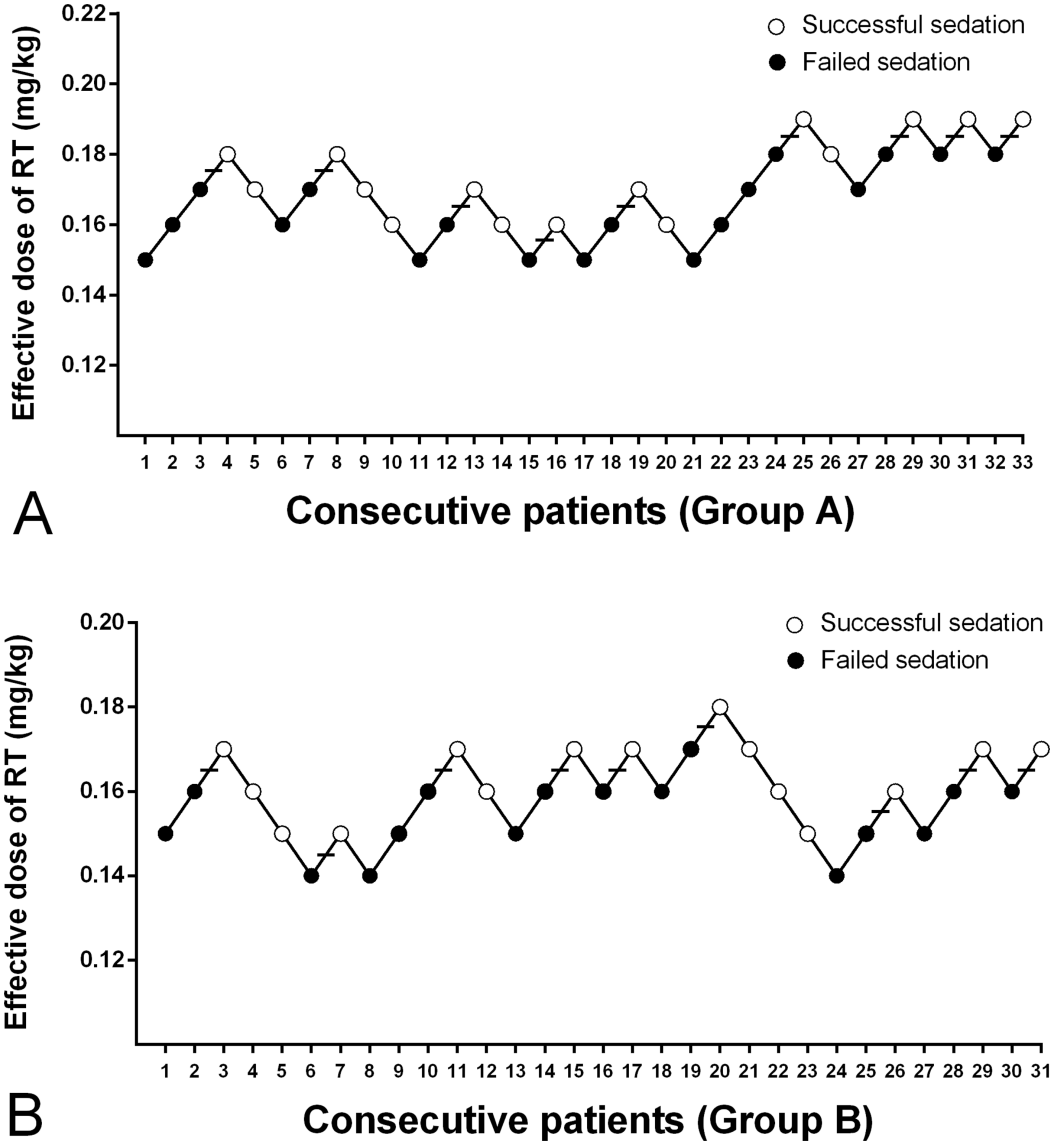
The dose of RT in all consecutive patients of **(A)** Group A and **(B)** Group B for inhibiting the gastroscopy reaction. Group A, patients aged 65–74 years; Group B, patients aged 75–89 years; Successful sedation (open circles); Failed sedation (filled circles); Horizontal bars indicate the crossover midpoints (positive–negative). RT, remimazolam tosilate.

**Figure 4:**
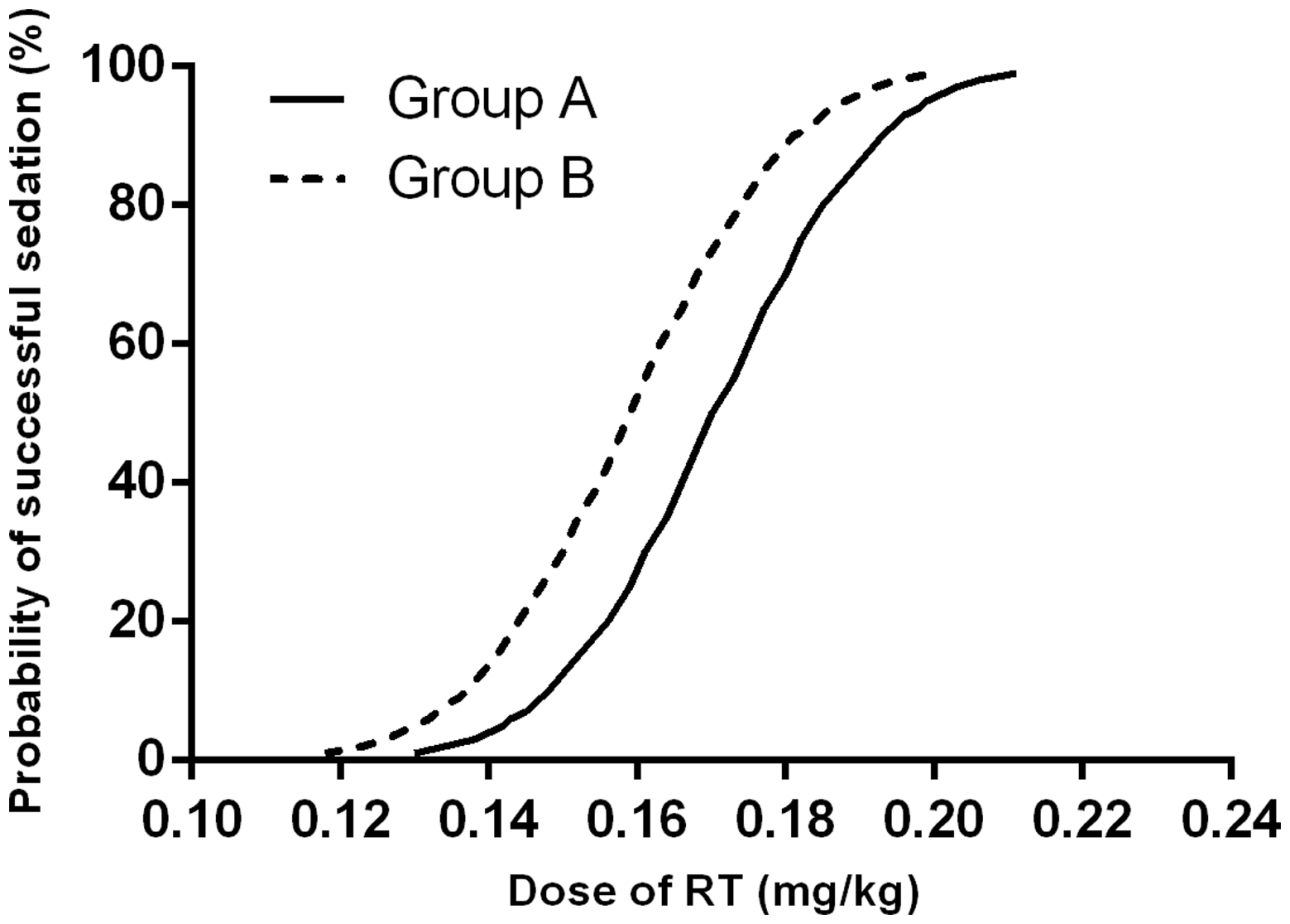
Dose–response curve from the probit analysis. Group A, patients aged 65–74 years; Group B, patients aged 75–89 years. RT, remimazolam tosilate.

**Figure 5:**
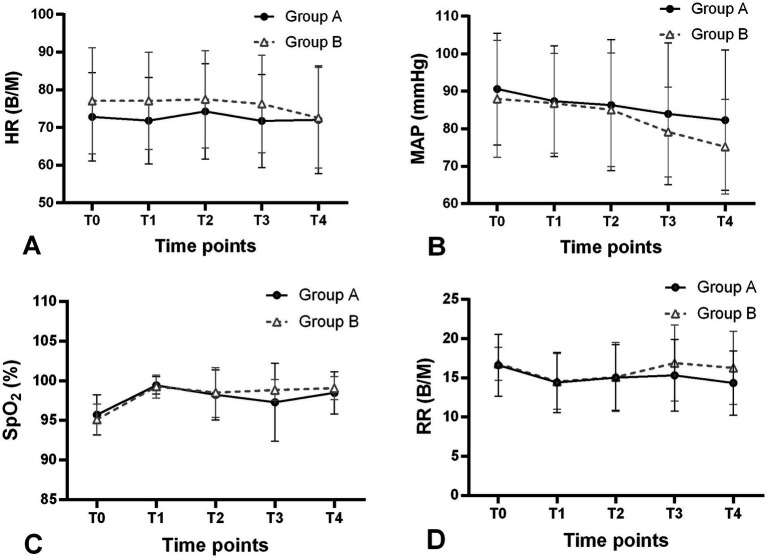
Changes in vital signs **(A)** HR, **(B)** MAP, **(C)** SpO_2_ and **(D)** RR over time in both groups during the trial. Group A, patients aged 65–74 years (filled circles); Group B, patients aged 75–89 years (open triangles); T0, on arrival to the endoscopy room; T1, after RT administration; T2, after endoscope insertion; T3, 5 min after start of gastroscopy; T4, end of gastroscopy. HR, heart rate; MAP, mean arterial pressure; SpO_2_, pulse oximetry; RR, respiratory rate; B/M, beats/min.

The modified UDM plots for the success and failure of gastroscopic sedation in the two groups were shown in Figures 3A,B, and nine crossover midpoints were reached by each group. The dose–response curve from the probit analysis of the RT dose and the probability of successful sedation in each group were shown in Figure 4. Of the 31 patients enrolled in Group A, 15 cases were negative response and 16 were positive response. Among the 30 patients enrolled in Group B, there were 15 negative and 15 positive response cases. The mean (95% CI) ED_50_ of RT for inhibiting the gastroscopy reaction calculated by the modified UDM in Group A (0.175 (95% CI, 0.166–0.183) mg/kg) was significantly higher than Group B (0.163 (95% CI, 0.156–0.169) mg/kg) (*p* = 0.03). It suggested that older elderly patients require less RT to achieve target sedation. To further verify the reliability of this result, we also used probit regression analysis, the mean (95% CI) ED_50_ and ED_95_ values estimated by the probit regression analysis were 0.170 (95% CI, 0.161–0.181) mg/kg and 0.199 (95% CI, 0.186–0.244) mg/kg in Group A, and 0.159 (95% CI, 0.149–0.169) mg/kg and 0.188 (95% CI, 0.175–0.232) mg/kg in Group B, respectively (Table 2). Various vital signs (including NBP, HR, SpO2 and RR) over time in both groups were recorded and shown in Figure 5.

**Table 2:** Effective dose of RT for inhibiting the gastroscopy reactions in Group A and Group B.

Effective dose	Estimation method	Group A (*n* = 31)	Group B (*n* = 30)	*p*
ED_50_ (mg/kg)	Modified UDM	0.175 (0.166–0.183)	0.163 (0.156–0.169)	0.03^∗^
	Probit regression analysis	0.170 (0.161–0.181)	0.159 (0.149–0.169)	
ED_95_ (mg/kg)	Probit regression analysis	0.199 (0.186–0.244)	0.188 (0.175–0.232)	

Data are presented as ED_50_ or ED_95_ with (95% CI). RT, remimazolam tosilate; Group A, patients aged 65–74 years; Group B, patients aged 75–89 years; UDM, Dixon’s up-and-down method; ED_50_, median effective dose; ED_95_, 95% effective dose; CI, confidence interval. *p < 0.05 was considered to indicate statistical significance.

The adverse events observed in this trial were recorded and shown in Table 3. No serious adverse events were observed throughout the trial, and there was no significant difference in the incidence of adverse events between the two groups. The proportions of hypotension cases were 10/31 (32%) in Group A and 12/30 (40%) in Group B, but only 2/31 (7%) in Group A and 1/30 (3%) in Group B needed ephedrine administered intravenously. A total of 5/31 (16%) of patients in Group A and 1/30 (3%) of patients in Group B experienced respiratory depression and required increased oxygen delivery, but only 1/31 (3%) of patients in Group A required assisted manual ventilation with 100% oxygen by jaw thrust maneuver, no one required removal of the gastroscope in this trial. Throughout the trial, neither group experienced adverse events such as injection pain, bradycardia, muscle rigidity or severe gastroscopy reactions such as nausea and vomiting.

**Table 3:** Adverse events of patients included.

Adverse events	Group A (*n* = 31)	Group B (*n* = 30)	*p*
Injection pain	0/31 (0%)	0/30 (0%)	-
Hypotension	10/31 (32%)	12/30 (40%)	0.529
Need ephedrine	2/31 (7%)	1/30 (3%)	1.000
Respiratory depression	5/31 (16%)	1/30 (3%)	0.195
Need 100% oxygen ventilation by jaw thrust maneuver	1/31 (3%)	0/30 (0%)	1.000
Need removal of gastroscope	0/31 (0%)	0/30 (0%)	-
Bradycardia	0/31 (0%)	0/30 (0%)	-
Muscle rigidity	0/31 (0%)	0/30 (0%)	-

Data are presented as *n*/total (%). Group A, patients aged 65–74 years; Group B, patients aged 75–89 years. *p<0.05 was considered to indicate statistical significance.

## 4 Discussion

The main risk of performing gastroscopy in elderly patients is the sedation used during the procedure; the increased age as an independent risk factor for many adverse events related to anesthesia has been proved by several studies, which significantly increases perioperative morbidity and mortality in elderly patients (15). As a consequence, age-related pharmacokinetics and individual administration during the sedation process in elderly patients should be given more attention. However, the bulk of the studies applied RT for gastroscopy focused on younger patients (16, 17). For instance, Borkett et al. (18) only administered doses of 0.10 mg/kg, 0.15 mg/kg, and 0.20 mg/kg of RT for gastroscopic sedation in patients aged 18–65 years, and the successful sedation rates were 32, 56 and 64%, respectively. A more rigorous grading of evidence is essential to ensure methodological transparency and strengthen the validity of conclusions in future studies. Few studies have focused on applying it to gastroscopic sedation in elderly patients of different ages, and the experience of the optimum and effective dose recommended is lacking for these patients. In our study, elderly patients were grouped into two age categories: 65–74 years and 75–89 years, to explore the effective dose of RT for inhibiting the gastroscopy reaction.

As already known, the modified UDM, as a sequential trial design, is able to improve the research efficiency, save the sample size and simplify the process of the trial (19–21). It is acceptable to the US regulatory agencies, published as a standard test method by the American Society for Testing and Materials and widely used to obtain the ED_50_ of drugs (13, 19). Basing the principle on the modified UDM, six crossover midpoints and six samples should be the minimum criteria (12, 20). Whereas a small sample size may lead to some limitations, at least 20–40 patients will provide stable estimates of the target dose for the most realistic scenarios (13, 22). As a consequence, nine crossover midpoints and the sample size of 31 (Group A) and 30 (Group B) patients were included in our study. Thus, our results are further substantiated by evidence and exhibit greater statistical significance. The preceding study reported that the parameter estimate of probit regression analysis was biased and the confidence interval of ED_50_ might be unrealistically narrow (23). Whereas, the ED_50_ values calculated by the modified UDM and probit regression analysis for each group in our study were quite similar, indicating that these results might be relatively credible, and the information may be useful for RT administration in elderly patients undergoing a gastroscopy procedure.

An earlier study by Liu et al. (24) administered 0.15 mg/kg of RT as the induction dose in patients undergoing colonoscopy aged 65–75 years. Considering this study and our previous pilot research, 0.15 mg/kg of RT was also set as the initial dose in the present study. A previous study reported the ED_50_ and ED_95_ of RT for general anesthesia induction were 0.088 and 0.118 mg/kg in patients aged 60–69 years, respectively; and 0.061 and 0.090 mg/kg in patients aged 70–85 years, respectively (25). While in our study, the ED_50_ and ED_95_ of RT were 0.175 and 0.199 mg/kg in patients aged 65–74 years, respectively; and 0.163 and 0.188 in patients aged 75–89 years, respectively. The reason for these differences might be that they aimed to induce (tracheal intubation) successfully at that point in time only, whereas the initial dose of RT in our study was designed to inhibit the gastroscopy reaction within the first 5 min since the start of gastroscopy. Owing to the majority of gastroscopy procedures being completed within 5 min, we explored the optimum initial dose of RT for successful sedation within the first 5 min since the start of gastroscopy. Moreover, the dose of sufentanil (0.1–0.3 μg/kg) in their study being three times the amount we administered. The potential interaction between remimazolam and opioids, as well as variations in dosages required to achieve target sedation levels, may influence the pharmacodynamic profile of remimazolam. Synergistic effects between the opioid (remifentanil and fentanyl) and remimazolam have been previously described, with the magnitude of the synergistic effects on sedation outcomes are being dependent on the opioid dose (26, 27). Coadministration of remifentanil had a pharmacodynamic interaction with remimazolam in MOAAS, BIS, and tolerance to laryngoscopy or tetanic Stimulation (28). The concurrent use of fentanyl reduces the dosage of RT required for sedation in a dose-dependent manner (29).

The preceding study showed that RT could produce a rapid onset and a short duration of action (30). Guo et al. (31) intravenously administered RT 0.15 mg/kg within 30 s and the mean (SD) time to loss of consciousness after administration was 20.6 (2.6) seconds, hence, the mean (SD) total induction time was about 50.6 (2.6) seconds. This is generally consistent with our median [IQR] induction time, 58 [50–76] seconds in Group A and 53 [46.8–62] seconds in Group B. It has previously been shown that the degree and duration of sedation with remimazolam were dose dependent, with the peak effect of sedation being observed approximately 1 to 4 min after the start of the infusion (32). This is similar to our finding, the sedation time of initial dose in Group A and Group B were 3.3 and 4.8 min, respectively. Besides, this study showed that the ED_50_ in Group A was higher than Group B, suggesting that the requirement of RT in the gastroscopy decreased with age and elderly patients should be considered to reduce the dosage of RT in clinical anesthesia.

Although remimazolam has slight suppressive effects on the cardiovascular system and a low incidence of hypotension (33–35), other studies have shown that the incidence of hypotension ranges from 25 to 37% (36, 37). This incidence of hypotension is consistent with ours (36% of overall 61 patients), and only 3/61 (5%) patients needed ephedrine administered intravenously. Moreover, no patient developed bradycardia and no intervention on HR was made with atropine. These demonstrate that RT has a relatively good stability for circulatory system. Similar to the findings by Borkett et al. (18), our results indicate that the incidence of respiratory depression was 16% (Group A) and 3% (Group B). Although 6/61 (10%) patients in this trial presented with respiratory depression, only 1/61 (2%) patients required a jaw thrust maneuver and assisted manual ventilation with 100% oxygen to treat hypoxemia, and the SpO_2_ quickly recovered to more than 90%. Zhang et al. (38) reported that 2.4% patients still experience injection pain when remimazolam was administered intravenously, but no injection pain occurred in our trial, which was likely due to the analgesic effect of sufentanil administered intravenously beforehand. In addition, no other adverse events and severe gastroscopy reactions (including muscle rigidity, nausea and vomiting) were observed throughout this trial. These results mentioned above suggest that RT had a good safety profile for elderly patients.

Several studies had indicated that elderly patients were more likely to have lower cardiac, renal, and other organic reserves, and the sensitivity to most intravenous hypnotic agents was also increased, elderly patients might be more likely to experience circulatory suppression after anesthesia/sedation (5, 15). Although various vital signs (including HR, MAP, SpO_2_ and RR) at T0-T4 time points were recorded, we did not conduct further statistical analysis. The reason was the inherent limitations of the modified UDM design: The sample size was small; The dose of RT for each patient was not fixed, which depended on the gastroscopy reaction of the previous patient. Even so, we could still roughly observe the gradual downward trends in MAP, reaching the bottom at the end of the gastroscopy in both groups. This might be due to the fact that RT had a slight suppressive effect on hemodynamics in elderly patients. Other vital signs of the two groups had fluctuated over time, but were generally stable.

Although our study may provide reference for sedation in elderly patients undergoing gastroscopy, there were still several limitations in this study. Firstly, this is a single-center study with a limited sample size, and the observed indicators are relatively simple, however, a cohort size of 20 to 40 patients is generally acceptable based on UDM methodology (13). Notably, this trial only explored the dose of RT for gastroscopic sedation in elderly patients, further studies are needed evaluating the effective dose of RT for underweight, obese, ASA physical status ≥III, elderly individuals at high risk, and the comparison between young patients and elderly patients. Moreover, only the gastroscopy procedure was included in this study, whether this protocol is feasible for colonoscopy is also worthy of further investigation.

## 5 Conclusion

In summary, our study indicates that RT is a relatively safe sedative hypnotic that can provide a suitable sedative effect with low incidence of adverse events in gastroscopy for the elderly. In clinical practice, ED_95_ would be required to have efficacy for 95% of the population, hence we recommend that the doses of RT to inhibit the gastroscopy reaction in elderly patients aged 65–74 years and 75–89 years are approximately 0.199 and 0.188 mg/kg, respectively.

## Data Availability

The raw data supporting the conclusions of this article will be made available by the authors, without undue reservation.
